# Psychopathic Traits in Childhood: Insights from Parental Warmth and Fearless Temperament via Conscience Development

**DOI:** 10.3390/brainsci11070923

**Published:** 2021-07-13

**Authors:** Laura López-Romero, Olalla Cutrín, Lorena Maneiro, Beatriz Domínguez-Álvarez, Estrella Romero

**Affiliations:** 1 Department of Clinical Psychology and Psychobiology, Universidade de Santiago de Compostela, 15782 Santiago de Compostela, Spain; olalla.cutrin@usc.es (O.C.); lorena.maneiro@usc.es (L.M.); beatrizdominguez.alvarez@usc.es (B.D.-Á.); estrella.romero@usc.es (E.R.); 2 Institute of Education and Child Studies, Leiden University, 2333 AK Leiden, The Netherlands

**Keywords:** psychopathic traits, childhood, fearlessness, parental warmth, conscience development

## Abstract

The role of psychopathic traits in predicting more serious and persistent patterns of child conduct problems has been well documented. The jointly presence of interpersonal (grandiose–deceitful), affective (e.g., callous–unemotional), and behavioral psychopathic traits (impulsive–need of stimulation) identifies a group of children at increased risk of psychosocial maladjustment. The present study aims to disentangle the underlying mechanisms by examining how early parenting (i.e., warmth) and child temperament (i.e., fearlessness) predict later psychopathic traits, via conscience development (CD). Data were collected in a large sample of children (*n* = 2.266; 48.5% girls), aged 3 to 6 at the onset of the study (*M*age = 4.25; *SD* = 0.91), who were followed up one and two years later. The results showed direct effects from fearlessness to interpersonal and behavioral psychopathic traits. Parental warmth, fearless temperament, and their interaction, predicted CD, which, in turn, showed a negative effect on psychopathic traits. The indirect effects indicated significant negative mediation effects of warmth through CD on psychopathic traits, which seem to be stronger when children present lower levels of fearlessness. Overall, these results contribute to better understand the development of child psychopathic traits and provide additional insight on effective strategies that will help to restrain the potential development of a high-risk profile in early childhood.

## 1. Introduction

Child conduct problems, involving a heterogeneous pattern of deviant behaviors such as aggression, rule-breaking, and oppositional or destructive behavior [1,2], have a negative impact on children’s socio-emotional development, as well as on family, school, and peer interactions, with an important cost for society [3,4]. In an attempt to further identify specific factors that may place a child at increased risk for being involved in an early-onset, severe and stable pattern of conduct problems, some authors have proposed the study of psychopathic personality at early developmental stages [5,6,7]. Psychopathic traits have been traditionally defined as a constellation of co-occurring interpersonal (i.e., grandiose–deceitful (GD)), affective (i.e., callous–unemotional (CU)) and behavioral/lifestyle (i.e., impulsive–need of stimulation (INS)) traits [8,9,10,11]. Over the past two decades, psychopathic traits have been consistently linked to more serious and persistent patterns of conduct problems and aggression, later antisocial behavior and delinquency, lower levels of social competence and prosocial behavior, and even to adult psychopathy [11,12,13], with some of these results being also replicated in early childhood [14,15,16]. Given the lasting negative consequences of early psychopathic traits, additional understanding on how they develop, by identifying potential underlying etiological mechanisms, is needed. Considering that children with conduct problems who also show psychopathic traits tend to benefit less from traditional interventions (e.g., parenting programs) [17,18,19], advancing our knowledge on this topic will shed new light on the development of effective strategies, tailored to the unique characteristics of children with psychopathic traits, for both prevention and intervention purposes.

### 1.1. Developmental Models of Psychopathic Traits

Psychopathic personality has been defined as a developmental disorder with its roots in early childhood [12,20]. The full array of interpersonal, affective, and behavioral psychopathic traits has been reliably identified at early developmental stages [9,21]. As was consistently observed, high levels of psychopathic traits identify a group of children at increased risk for more serious and persistent problems, showing a closer association with long-lasting behavioral and psychosocial disturbances, as well as with distinctive etiological mechanisms (see the compendious reviews [11,12]).

Comprehensive developmental models on the etiology and later development of psychopathic traits have suggested that certain temperamental styles, such as behavioral disinhibition or fearlessness, are linked with problems in conscience development (CD), these associations being critical for understanding the emergence of psychopathic traits (see [22]). The construct of conscience, refers to the development, maintenance, and application of generalizable, internal regulators of one’s behavior [23]. While the broader concept is multifaceted, comprising diverse affective (e.g., moral emotions), regulatory (e.g., self-control capacities), motivational (e.g., responsiveness to socialization) and cognitive (e.g., moral cognitions) components and processes [24,25], conscience has been often defined by guilt and empathy [26]. These moral emotions represent, as well, two of the hallmarks of the construct of psychopathy, and play an important role in a child’s development by promoting prosocial development whilst restraining antisocial behavior. From this theoretical perspective, problems in CD would be largely due to problems in the development of guilt and empathy, which in turn would be influenced by certain temperamental styles, including fearlessness, insensitivity to punishment or low responsiveness to cues of distress [27,28]. More specifically, children characterized by a fearless and disinhibited temperament tend to seek out novel situations to test limits, and usually do not fear the consequences of misbehavior, which places them at greater risk to engage in dangerous activities [29]. This temperamental style, characterized by a poor autonomic arousal in the presence of punitive stimuli, contributes, across development, to failure in interiorizing parental and societal norms, rules and regulations, restraining the appearance of internal states and emotions that would ensure compliance and commitment to the norms and demands that come from the environment (i.e., the development of moral and emotional consciousness) [23,30]. This multiple chain of deficits could also be on the basis of the development of psychopathic traits [31,32]. In support of this assumption is the evidence revealing that children high on psychopathic traits also tend to show a fearless temperament [28,33,34], and a pronounced lack of remorse and empathy [35]. As suggested by Blair and Cipolotti [36], a general emotional impairment may affect the development of moral emotions, eventually leading to dishonest and careless behavior, which is also a core element in the psychopathy definition [37].

Notwithstanding the importance of temperamental factors in the development of psychopathic traits, it should be noted that they are supposed to be largely due to biological deficits and, therefore, might be difficult to avoid or prevent. When practical implications are prioritized, the identification, assessment, and management of those factors able to enhance, maintain, or restrain such developmental process gain more prominence. In this regard, the role of parenting practices in the development of psychopathic traits has been evidenced as particularly influential (see [38]), with parental warmth predicting a reduction, whilst parental harshness favoring an increase, in overall psychopathic [39] and more specific CU traits [40,41,42,43] across childhood and adolescence. Parenting practices have also shown a clinical value not only in reducing problematic behavior in children with high psychopathic traits [44], but also in favoring a significant reduction in all affective, interpersonal, and behavioral features of psychopathic personality [45].

In this context, after evidencing that not all children showing a fearless and uninhibited temperament will invariable manifest deficits in moral emotions, most theories of CD have also considered the role of parenting, which interacts with a child’s temperament in a complex dynamic process [46]. In this regard, it has been suggested that parenting practices, particularly those relying on parental warmth, affection, and other positive qualities, may be especially influential for CD in fearless children [46,47]. Similar interactions have been replicated in current developmental models on the etiology of CU traits, suggesting that highly positive parenting buffered the risk that fearlessness posed to the development of CU behaviors [48,49].

### 1.2. The Present Study

Based on the foregoing, it can be suggested that child temperament interacts with parental practices to increase or buffer the risk for later psychopathic traits [50], with these effects being potentially driven by changes in CD. Nevertheless, it should be noted that most of the aforementioned literature has focused on the development of CU traits, which represents the affective dimension of the psychopathy construct. Even considering that research from the CU perspective has provided a great knowledge in the field, studying psychopathic personality from a multidimensional perspective (i.e., including all interpersonal, affective, and behavioral traits) has proven to be effective in identifying children at increased risk for later maladjustment, even when compared to children just high on CU traits [16,51,52]. Therefore, if we aim to better identify this high-risk profile early in development, psychopathic personality, with all its dimensions, should be taken into account [53,54]. Current research on the multidimensional construct in young children has basically examined its internal structure and predictive value, with studies aimed at identifying distinctive etiological mechanisms mainly conducted in older samples (see [11]). In this regard, it is important to test whether previous findings on CU traits can be extrapolated to other psychopathy dimensions or, in turn, whether a combination of high interpersonal, affective, and behavioral psychopathic traits may identify a distinctive etiological subgroup of children at increased risk for later maladjustment. It is also important to examine these questions in early childhood because this is a key period for developmental foundations of empathy and conscience [47], and when severe trajectories of child conduct problems emerge [55]. Answering these questions will help to clarify the mechanisms underlying the development of psychopathic traits, with potential practical implications relevant to assessment, diagnostic classification, and tailored interventions.

The main purpose of the current study was, therefore, to unravel the etiological mechanisms leading to psychopathic traits in childhood by testing a developmental model where parenting practices and fearless temperament interact to predict psychopathic traits, via CD. Since guilt and empathy can be identified early in life, and because they play an important role in the development of psychopathic traits [9,35], the current study only focused on the emotional components of CD. Furthermore, because parental warmth has proven to be relevant in the development of both CD and psychopathic traits [38,47,56], being also an important target for recent adaptations to intervene with children high on psychopathic traits [57], it was included as a measure of parenting. We expected that both parental warmth and fearlessness would drive effects on psychopathic traits via CD. Based on previous research, we hypothesized that parental warmth would have effects on both CD and psychopathic traits, particularly at lower levels of fearlessness [46].

## 2. Materials and Methods

### 2.1. Participants

The Estudio Longitudinal para una Infancia Saludable (Longitudinal Study for a Healthy Childhood; (ELISA)) is a prospective longitudinal study conducted in Galicia (NW Spain) with the aim of better understanding the behavioral, emotional, personality, and psychosocial development from early childhood to adolescence. For the purposes of the current study, parent-reported information collected in the first three waves of study were included in the analyses. Data collection started when children were in preschool (i.e., children aged 3 to 6 years old), encompassing children born in 2011–2013. Only children with available data in some of the main study variables, namely fearlessness, parental warmth, guilt and empathy, and psychopathic traits, were included in the present study, resulting in an initial sample of 2266 children (48.5% girls), who were on average 4 years old (*M*age = 4.25; *SD* = 0.91). A total of 72 public (79.2%), charter (18.1%), and private (2.8%) schools participated in the study, which were located in predominantly working-class communities, with no diversity in terms of ethnicity (93.9% of children were Spanish). Regarding children’s family background, 23.7% of mothers and 39.8% of fathers completed compulsory education, 47.4% and 31.2% completed higher education, and 28.9% and 29% completed vocational training studies.

Two follow-ups were conducted within one-year intervals. Thus, the first follow-up (T2) was conducted one year after the initial wave of data collection and the second follow-up (T3) was conducted two years after the first wave of the study. The level of attrition is considered adequate, since 88.6% of respondents who participated in T1 participated in T2, and 76.3% of respondents who participated in T1 also participated in T3. As commonly observed in longitudinal studies, attrition was derived from death or frailty, withdrawal, lack of success in additional contacts for a follow-up survey, or by non-returning a survey by some participants [58]. Comparisons between participating families and families who dropped out of one wave of the study revealed no significant differences in terms of age *F*_(2248)_ = 2.51, *p* = 0.082, and initial (T1) levels of conduct problems *F*_(2227)_ = 0.30, *p* = 0.741. However, differences in terms of gender and SES were found between groups. Specifically, there was a higher proportion of boys in families who dropped out of one wave of the study (*χ*^2^_(2)_ = 11.88, *p* < 0.01); whereas higher levels of SES were found in families who participated in all three waves of the study (*F*_(2249)_ = 16.27, *p* < 0.001), a result consistently found in previous longitudinal research [58]. Even though, in longitudinal studies spanning different developmental periods, one may expect other developmental variables being also affecting participation rates [59].

### 2.2. Measures

To assess the intended constructs, measures specifically developed for being used with preschool children were selected. Moreover, the use of validated measures with a Spanish version were prioritized. In case it was not available at the time of data collection, items were adapted and translated by the research group, according to the standard guidelines for translation and adaptation of instruments [60].

*Covariates.* In addition to the gender of the child (1 = boy; 2 = girl), we accounted for the socioeconomic status (SES) of the family in the first wave of study. The SES variable was created by combining the scores on a set of items related to the socioeconomic background of the family (i.e., academic level of the mother and academic level of the father, monthly income, and parent’s concerns about the family economic situation).

#### 2.2.1. Baseline Variables (T1)

*Parental warmth*. A parent-reported scale based on the Warmth subscale from the Child Rearing Scale (CRS) [61,62], included in previous studies with preschool children [63], was used to assess the levels of parental warmth. This scale is composed of 6 items (α = 0.82; e.g., “You express affection by hugging, kissing, and holding your child”, “You have warm, close times together with your child”), scored in a 5-point scale (1 = *never* to 5 = *very often*).

*Fearlessness*. The level of child’s fearlessness was reported by parents through a scale consisting of six items (α = 0.85; e.g., “He/she does not seem to be afraid of anything”, “He/she does not seem to be afraid when someone is trying to frighten him/her”), developed for being used from age three, and used in previous studies, including the ELISA project [9,16]. Parents scored each item on a four-point scale, ranging from 1 (*Does not apply at all*) to 4 (*Applies very well*).

#### 2.2.2. Mediating Variable (T2)

A latent variable of CD was defined by the composite score of two observed variables, namely guilt and empathy.

*Guilt*. A parent-reported scale composed of 5 items (α = 0.64; e.g., “Doesn’t act very upset when he/she has done something wrong”, “He/she seems to feel guilty after breaking a rule”) was used to measure the level of guilt displayed by the child. The items were scored in a 7-point scale from 1 (*Definitely false*) to 7 (*Definitely true*). This scale was adapted from the guilt/shame scale that was developed and included in the long form of the Children’s Behavior Questionnaire (CBQ) [64,65] as an additional measure to assess specific social behavior patterns. The items were adapted from the Spanish (European) version of the standard CBQ retrieved, under request, from the author’s official website. The guilt/shame scale was originally composed of 14 items [64], however, for the purposes of the current study, only the items corresponding to the facet of guilt were considered.

*Empathy*. A parent-reported scale adapted from the Griffith Empathy Measure (GEM) [66], a measure intended to assess empathy from preschool years onwards, was used to assess child’s empathy. Parents rated six items, adapted and translated by the research team, referring to cognitive empathy (3 items; e.g., “Doesn’t seem to understand why people get upset”, “Rarely understands why other people cry”) and affective empathy (3 items; e.g., “Feels sad when other children or people are upset”, “Feels happy when someone else is happy”), in a four-point scale from 0 (*Totally disagree*) to 3 (*Totally agree*). The global empathy score was used in the present study (α = 0.67).

#### 2.2.3. Longitudinal Outcomes (T3)

*Psychopathic traits*. The parent-reported version of the Spanish Child Problematic Traits Inventory (CPTI) [9,67] was used for the assessment of child’s psychopathic traits. The scale is composed of 28 items, specifically developed to be used in children from age 3, and grouped in three subscales: eight items to measure the interpersonal or grandiose–deceitful (GD) psychopathy component (α = 0.83; e.g., “Thinks that he or she is better than everyone on almost everything”), 10 items to measure the affective or callous–unemotional (CU) psychopathy component (α = 0.88; e.g., “Never seems to have bad conscience for things that he or she has done”), and 10 items to measure the behavioral or impulsive–need of stimulation (INS) psychopathy component (α = 0.86; e.g., “Provides himself or herself with different things very fast and eagerly”). Parents rated the CPTI items in a response scale ranging from 1 (*Does not apply at all*) to 4 (*Applies very well*).

### 2.3. Procedure

This study was approved by the Bioethics Committee at the Universidade de Santiago de Compostela, and the Spanish Ministry of Economy and Competitiveness. A total of 126 public, charter and private schools were initially contacted in order to ask for potential collaboration. The contacts were initially conducted by phone, and information letters were subsequently sent by email. If a school accepted to take part in the study, families were contacted and invited to participate via information letters and group meetings in the schools. An active consent form was filled out by families (i.e., mother, father, or main caregiver) for each child who participate in the study. The informed consents were collected by preschool teachers, who handed out the information to the parents. In all the waves of study, participants were given a month to fill out the questionnaires. After that period, some reminders were sent to those who were late, firstly by the preschool teacher and then directly by the ELISA staff via email. Families did not receive any monetary compensation for their participation in the study. Nonetheless, as a reward for their participation, all the schools received a set of educational games for preschoolers in T1, whilst both families and schools participated in a draw of several sets of books and educational games, valued between EUR 50 and 100, at the end of the third wave data collection (T3).

### 2.4. Data Analyses

**Figure 1:**
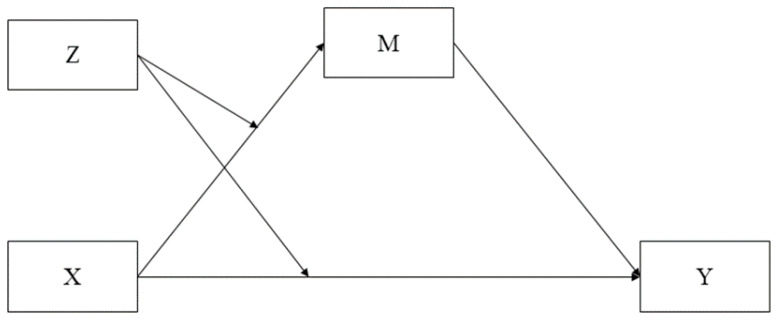
Conceptual model of conditional indirect effects. Model representing X as a predictor, Z as a moderator, M as a mediator, and Y as a dependent variable.

Firstly, descriptive statistics and zero-order correlations among all the study variables were analyzed. To test the conditional indirect effects of parental warmth, fearlessness, and CD on psychopathic traits, an SEM model was conducted in Mplus 7.4 [68]. The model included parental warmth as the predictor, fearlessness as the moderator (i.e., low, moderate, and high), the interaction between parental warmth and fearlessness, CD as the mediating variable, and the three psychopathic traits (i.e., GD, CU, and INS) as endogenous variables (see the conceptual model depicted in Figure 1). The predictor variables were mean-centered prior to the creation of the interaction term (i.e., warmth × fearlessness) in order to account for multicollinearity among variables [69]. The model was estimated using Full Information Maximum Likelihood (FIML), considered the least biased method of estimating missing information when indicators are missing at random [70]. Model fit was assessed using the root mean square error of approximation (RMSEA), standardized root mean squared residual (SRMR), comparative fit index (CFI), and the Tucker–Lewis index (TLI). According to Hu and Bentler’s [71] suggestions, RMSEA and SRMR values lower or equal to 0.05, and TLI and CFI values of 0.95 or higher were considered indicators of a good model fit, whereas RMSEA and SRMR values smaller than 0.08, and TLI and CFI larger than 0.90 indicated an adequate model fit.

## 3. Results

### 3.1. Descriptive Statistics and Zero-Order Correlations

Descriptive statistics and bivariate correlations among all the study variables are displayed in Table 1. Parents reported very high levels of parental warmth and high levels of guilt and empathy in their children, as indicated by means very close or slightly close to the maximum rating. Parents reporting their children showed low to mid-levels of fearlessness and impulsive (INS) traits and very low levels of interpersonal (GD) and affective (CU) psychopathic traits. Parental warmth and children’s empathy and guilt were significantly and positively related, whereas they were negatively related to fearlessness and all the psychopathic traits. Fearlessness and all the psychopathic traits showed a significant positive correlation.

### 3.2. Conditional Indirect Effects

**Figure 2:**
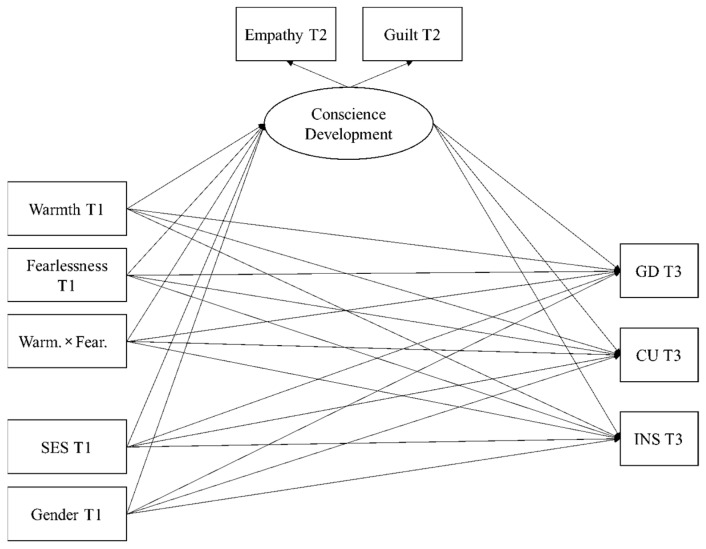
Structural equation model computed to test conditional indirect effects in the current study. The model considers parental warmth as the predictor, fearlessness as the moderator, conscience development as the mediator; Grandiose–Deceitful (GD), Callous–Unemotional (CU), Impulsive–Need of Stimulation (INS) traits as the dependent variables; and socioeconomic status (SES) and gender as the control variables.

Figure 2 shows the structural equation model (SEM) computed to test the conceptual model of conditional indirect effects on psychopathic traits (i.e., GD, CU, INS) at T3. This model included parental warmth as the predictor and fearlessness as the moderator, both of them measured at T1; and CD as the mediator, which is a latent variable created from empathy (λ = 0.55, *p* < 0.001) and guilt (λ = 0.63, *p* < 0.001), both measured at T2. This model showed an adequate model fit, χ^2^_(6)_ = 48.381, *p* < 0.001; CFI = 0.98; TLI = 0.90; RMSEA = 0.06; SRMR = 0.01. All the psychopathic traits are significantly correlated in the SEM (r_GD-CU_ = 0.45, *p* < 0.001; r_GD-INS_ = 0.42, *p* < 0.001; r_CU-INS_ = 0.30, *p* < 0.001).

**Figure 3:**
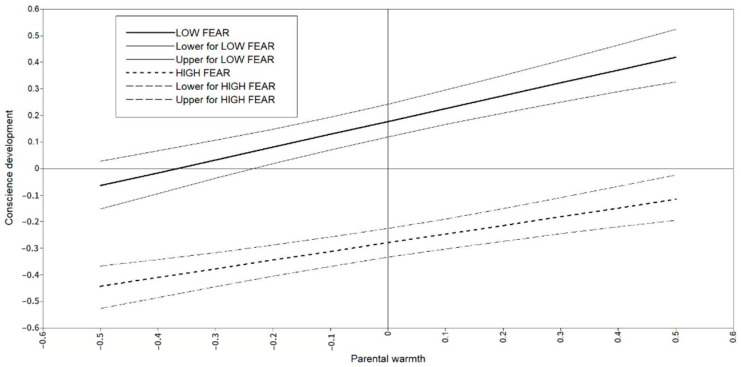
Estimated conscience development at T2 by the interaction of parental warmth and fearlessness at T1.

The standardized results regarding the direct relationships modeled in Figure 2 are shown in Table 2. Control variables (SES and gender) significantly predicted CD, with higher levels of SES and being female predicting higher levels of CD in children. Parental warmth significantly and positively predicted CD; i.e., higher levels of warmth predicted higher levels of CD in children. Children’s fearlessness significantly predicted CD negatively, and GD and INS traits positively; that is, higher levels of fearlessness predicted lower levels of CD and higher levels of interpersonal and behavioral traits in children. For its part, CD significantly and negatively predicted all three psychopathy dimensions in children. Finally, a negative interaction between parental warmth and fearlessness significantly predicted CD. The interaction term reflects that the positive relationship of warmth with CD tends to be stronger when children show low levels of fearlessness (see Figure 3). However, the very low magnitude of the interaction prevents us from clearly visualizing the differential tendency on the slopes.

The conditional indirect effects computed in the model can help to interpret all these associations, which can be summarized in the following terms. Warmth directly predicted CD (mediator) which directly predicted all psychopathic traits (i.e., potential mediation effects), while the relationship between warmth and CD was moderated by fearlessness (i.e., potential moderated mediation effects). Because the direct effects of warmth on psychopathic traits were not moderated by the level of fearlessness, mediated moderation effects were discarded; i.e., regardless of the level of fearlessness, parental warmth is not directly related to GD, CU, and INS. As shown in Table 3, and in line with the interaction plot displayed in Figure 3, the unstandardized results of indirect effects indicated the significant presence of negative mediation effects of warmth through CD on psychopathic traits, which seem to be stronger when children present lower levels of fearlessness.

Lastly, because fearlessness has been traditionally related with both CD and psychopathic traits [28,47], and taking into account the very low moderation effect previously observed, the potential mediation effects of fearlessness on children’s psychopathic traits through CD was also tested. As reported in Table 2, fearlessness significantly negatively predicted CD (*β* = −0.33, *p* < 0.001; path a), which, in turn, significantly predicted GD (*β* = −0.39, *p* < 0.001; path b_1_), CU (*β* = −0.64, *p* < 0.001; path b_2_), and INS (*β* = −0.32, *p* < 0.001; path b_3_). These relationships might be indicative of mediation effects (a * b_i_) of fearlessness through CD on GD (*β* = 0.13), CU (*β* = 0.21), and INS (*β* = 0.11). The Sobel test indicated that all these mediation effects were statistically significant (Z_GD_ = 7.43, *p* < 0.001; Z_CU_ = 7.99, *p* < 0.001; Z_INS_ = 7.32, *p* < 0.001).

## 4. Discussion

Psychopathic personality has emerged as an important construct for better understanding child conduct problems [72]. Through a burgeoning line of research, psychopathic traits have been linked with a large set of problematic behaviors and negative outcomes from early childhood onwards [11,12]. Although most researchers usually define psychopathic personality as a constellation of co-occurring interpersonal, affective and behavioral traits [8,9], research conducted in childhood has mainly focused on the role of the affective dimension (i.e., CU traits), with the broader construct of psychopathy being still underrepresented [53,54,73]. This is particularly true as pertaining the etiological mechanisms leading to the development of psychopathic traits at early developmental stages. Advancing this knowledge might offer great utility to optimize our research and clinical practice [54], delving into more sophisticated developmental models of psychopathic personality and conduct problems. This would inspire the design of new intervention strategies that may lead to prevention and reduction of psychopathic traits and, therefore, to restrain the development of severe and persistent patterns of problematic behavior.

### 4.1. Fearlessness, Warmth, and Conscience Development: Unraveling the Paths to Psychopathic Traits

From a developmental model that posits the effect of temperamental variables (i.e., fearlessness) and parenting practices (i.e., parental warmth) in the development of psychopathic traits, the current study intended to account for person-by-context interactions that are supposed to interplay in a dynamic process that could influence the basis of both CD [46] and, subsequently, psychopathic traits [49]. Overall, results revealed that parental warmth did not have direct effects on psychopathic traits, although there would be indirect effects, totally mediated by CD, on all three psychopathy dimensions. More specifically, current results suggested that higher levels of parental warmth in preschool years (T1; ages 3 to 6) would predict higher levels of CD one year later (T2; ages 4 to 7), which, in turn, would potentially restrain the development of psychopathic traits at T3 (ages 5 to 8). Even considering that all indirect effects were significant irrespective of the level of fearlessness, a marginal interaction effect showed that higher levels of parental warmth predicted increases in CD particularly at lower levels of fearlessness. This result largely converges with previous studies on the developmental basis of CD, indicating that positive parenting practices (i.e., parental warmth), would be particularly influential for CD in fearless children [46,47]. Similar findings, yet revealing the inverse pattern, were shown in previous research examining the role of fearlessness and parenting practices (i.e., parental warmth, and parental harshness) in the development of CU traits. Thus, in a sample of low-income boys, child fearlessness only predicted early CU traits in the context of low positive parenting [49], a result also replicated in an adoption design that showed that early fearlessness, which was mainly inherited from biological mothers, was predictive of CU traits when adoptive parents showed lower levels of positive parenting [48].

Interestingly, our results evidenced the same conditional effects for all psychopathy dimensions, suggesting that previous findings linked to CU traits could be expanded, at least as pertaining to this model, to all three psychopathy dimensions. Although preliminary, this is an important result since most of previous research has examined the effects of both fearlessness and parental warmth on CU traits, without accounting for the potential shared effects with both GD and INS traits. It should be noted that current results showed direct effects of fearlessness on GD and INS traits, but not the expected direct effects on CU traits [28,34]. Although this result might be initially unexpected, it could largely converge with previous research, with the effects of fearlessness on CU traits being potentially mediated by CD. As was previously mentioned, empathy and guilt have been defined as components of conscience [26], as well as two of the hallmarks of CU traits [35]. Additionally, temperamental features related with CU traits, such as fearlessness, have been considered risk factors for impairments in empathy and, therefore, in the normal development of conscience and morality in children [27,28]. Based on the foregoing, a fearless temperament would be linked to CD by underpinning potential deficits in the development of empathy and guilt, which in turn, would be predictive of later CU traits.

### 4.2. Theoretical and Practical Implications

From a developmental psychopathology perspective, the development of a psychopathic personality is usually viewed as a dynamic, changing, and ongoing process [22]. Which factors are influencing its development, being capable to potentiate, maintain or alter its course, is still an ongoing challenge in this field. The basic developmental principle of *equifinality* is likely to be pertinent in the etiological mechanisms involved in psychopathic personality. Thus, it is not expected that a single factor—at the genetic, neurobiological, neurocognitive, developmental or environmental level—would act in an isolated way in the etiology and developmental pathway of psychopathic traits [74]. Dynamic interactive processes are probably behind the developmental underpinnings of the construct, being the associations between the temperament and environment complex and, probably, in a bidirectional way [40,63]. Moreover, it should be noted that the links between parenting practices and psychopathic traits could reflect gene–environment interactions [75], with psychopathic traits being potentially rooted via heritable patterns [76], whereas parenting would play a role as a potential environmental-mediated predictor of psychopathic traits [49]. Even considering the progress made in linking CD theories with the development of psychopathy traits, new advances are required to improve developmental models of psychopathic personality, integrating all the most relevant knowledge in a coherent paradigm that may help to further understand the early underpinnings and later developmental patterns of psychopathic traits. In this regard, Waller and Wagner [77] have proposed the Sensitivity to Threat and Affiliative Reward Model (STAR) as a comprehensive model to delve into the etiology of CU traits. It posits that both fearless temperament and low affiliative reward would be the two psychobiological and mechanistic precursors to CU traits, with their interaction being uniquely predictive of CU traits when compared to GD and INS [78]. Assuming the promising value of the STAR model for the development of CU traits, new efforts are needed to disentangle the mechanisms leading to both GD and INS and, even more interesting, to test whether these specific mechanisms are also central when all psychopathic traits are present.

Finally, current results would also derive some practical implications. Since psychopathic traits seem to be identifiers of youths within serious and long-standing pathways of problematic behavior, they should be primarily identified in clinical settings. To maximize results, these programs should be tailored to the unique characteristics that define this specific group (e.g., remorseless, lack of empathy, manipulation), and should include those factors that have been proven to be potential mechanisms of change in psychopathic personality (e.g., parental warmth). Some promising results from the applied context have shown that focusing on improving parental warmth, whilst declining inconsistent and coercive parenting, may have clinical value not only in reducing problematic behavior in children with high psychopathic traits, but also in favoring a significant reduction in all affective, interpersonal, and behavioral features of psychopathic personality [45,57]. Based on current findings, these effects could be also strengthened by stimulating the development of conscience through specific socioemotional training, favoring emotion recognition and affective responsivity [79].

### 4.3. Limitations and Future Lines of Research

To our knowledge, this is one of the first studies linking the developmental model of CD to psychopathic personality, accounting for all its dimensions. It involves a large sample of children from a prospective longitudinal study, conducted across two years, and starting at the preschool years, which is considered a key stage in the development of empathy and conscience [47]. Even though, there are some limitations that should be acknowledge in order to address future research. First, we only relied on parents’ reports, which could have inflated some effects due to shared method variance. Second, we only focused on the affective component of conscience (i.e., moral emotions), making it necessary to further examine how other dimensions (e.g., moral reasoning), as well as the whole construct, are shaped from early socialization experiences in interaction with temperamental styles. From this chain of influences, it would be interesting to further examine how they finally contribute to the development of psychopathic traits and related disruptive behavior. Third, only fearlessness and parental warmth were included as predictors, making difficult to elaborate a comprehensive developmental model that would require some additional temperamental (e.g., affiliative reward), cognitive (e.g., emotion recognition) and environmental variables (e.g., parental harshness), including the quality of parent–child interactions, to delineate all the process and mechanisms leading to the construction of self and, in turn, to the development of psychopathic traits. Fourth, current results showed that gender may account for some of the effects, suggesting that additional research should take a gender perspective in order to examine whether there might be differences across groups in developmental mechanisms of psychopathic traits, which may also vary across development. Fifth, although a prospective longitudinal design was used, one might prevent to derive causal effects that should be examined in future research accounting for additional person-by-context interactions. Finally, additional studies addressing all psychopathy dimensions are particularly needed to further understand the etiological mechanisms underlying the development of specific psychopathic traits, and overall psychopathic personality, from early childhood onwards.

## 5. Conclusions

Current results allow strengthening developmental models of psychopathic traits, with parenting practices based on warmth and affection emerging as environmental mechanisms able to prompt changes in psychopathic traits across development [39], particularly at lower levels of fearlessness, and via CD. In this regard, it could be suggested that, whereas temperamental mechanisms (i.e., fearlessness) may underpin the most relevant features of psychopathic personality, environmental factors in general, and parenting practices in particular, would be able to either enhance or hinder their development across their lifespan [11], with CD as a potential mediator of these effects. It reinforces the possibility of designing new intervention strategies specifically tailored to the unique features of children with psychopathic traits, and the mechanisms able to drive some changes across development, leading to also prevent the development of serious patterns of problematic behaviors.

## Figures and Tables

**Table 1:** Descriptive statistics and bivariate correlations among the study variables.

	M (SD)	Range	1	2	3	4	5	6	7
1. Warmth T1	4.70 (0.38)	1–5	1						
2. Fearless T1	1.79 (0.66)	1–4	−0.05 *	1					
3. Guilt T2	5.08 (1.10)	1–7	0.17 ***	−0.23 ***	1				
4. Emp T2	2.19 (0.47)	0–3	0.10 ***	−0.19 ***	0.35 ***	1			
5. GD T3	1.37 (0.45)	1–4	−0.11 ***	0.22 ***	−0.33 ***	−0.16 ***	1		
6. CU T3	1.32 (0.43)	1–4	−0.14 ***	0.24 ***	−0.40 ***	−0.37 ***	0.59 ***	1	
7. INS T3	2.06 (0.60)	1–4	−0.10 ***	0.40 ***	−0.29 ***	−0.22 ***	0.53 ***	0.48 ***	1

Note: Fearless—fearlessness; Emp—empathy; GD—grandiose–deceitful; CU—callous–unemotional; INS—impulsive–need of stimulation; T1—wave 1; T2—wave 2; T3—wave 3. *** *p <* 0.001. * *p <* 0.05.

**Table 2:** Standardized direct effects from the SEM testing the conceptual model of conditional indirect effects.

	CD T2	GD T3	CU T3	INS T3
	β	β	β	β
SES T1	0.08 **	−0.00	−0.02	−0.03
Gender (0-male, 1-female) T1	0.12 ***	0.01	−0.02	−0.03
Warmth T1	0.22 ***	−0.02	0.02	−0.01
Fearless T1	−0.33 ***	0.08 **	0.01	0.28 ***
Warmth × Fearless T1	−0.04 *	0.01	−0.03	0.02
CD T2	-	−0.39 ***	−0.64 ***	−0.32 ***

Note: SES—socio-economic status; Fearless—fearlessness; CD—conscience development; GD—grandiose–deceitful; CU—callous–unemotional; INS—impulsive–need of stimulation; T1—wave 1; T2—wave 2; T3—wave 3. Warmth and fearlessness at T1 are mean-centered variables. Conscience development is a latent variable created by the observed variables guilt and empathy at T2. *** *p <* 0.001. ** *p <* 0.01. * *p <* 0.05.

**Table 3:** Unstandardized indirect mediation effects of parental warmth (T1) on psychopathic traits (T3) through conscience development (T2) moderated by the level of fearlessness (T1).

Warmth through CD with	GD T3		CU T3		INS T3	
	Est.	95% CI	Est.	95% CI	Est.	95% CI
Low fearless.	−0.12 ***	−0.16, −0.09	−0.18 ***	−0.23, −0.14	−0.13 ***	−0.17, −0.09
Medium fearless.	−0.10 ***	−0.14, −0.08	−0.15 ***	−0.19, −0.13	−0.11 ***	−0.14, −0.09
High fearless.	−0.08 ***	−0.10, −0.06	−0.12 ***	−0.15, −0.10	−0.09 ***	−0.12, −0.07

Note: Fearless—fearlessness. CD—conscience development. GD—grandiose–deceitful. CU—callous–unemotional. INS—impulsive–need of stimulation. CI—confidence interval. *** *p* < 0.001.

## Data Availability

Data presented in this study are available upon request to the corresponding author.

## References

[1] Blair R.J.R., Leibenluft E., Pine D. (2014). Conduct disorder and callous-unemotional traits is youth. N. Engl. J. Med..

[2] Frick P.J., Matlasz T., Martel M. (2018). Disruptive, impulse-control, and conduct disorders. Developmental Pathways to Disruptive, Impulse-Control and Conduct Disorders.

[3] Reef J., Diamantopoulou S., van Meurs I., Verhulst F.C., van der Ende J. (2011). Developmental trajectories of child to adolescent externalizing behavior and adult DSM-IV disorder: Results of a 24-year longitudinal study. Soc. Psychiatry Psychiatr. Epidemiol..

[4] Rivenbark J.G., Odgers C.L., Caspi A., Harrington H., Hogan S., Houts R.M., Poulton R., Moffitt T.E. (2018). The high societal costs of childhood conduct problems: Evidence from administrative records up to age 38 in a longitudinal birth cohort. J. Child Psychol. Psychiatry.

[5] Frick P.J. (2012). Developmental pathways to conduct disorder: Implications for future directions in research, assessment, and treatment. J. Clin. Child Adolesc. Psychol..

[6] Pardini D., Frick P.J. (2013). Multiple developmental pathways to conduct disorder: Current conceptualizations and clinical implications. J. Can. Acad. Child Adolesc. Psychiatry.

[7] Waller R., Hyde L.W., Grabell A.S., Alves M.L., Olson S.L. (2015). Differential associations of early callous-unemotional, oppositional, and ADHD behaviors: Multiple domains within early-starting conduct problems?. J. Child Psychol. Psychiatry.

[8] Cooke D.J., Michie C. (2001). Refining the construct of psychopathy: Towards a hierarchical model. Psychol. Assess..

[9] Colins O.F., Andershed H., Frogner L., Lopez-Romero L., Veen V., Andershed A.K. (2014). A New Measure to Assess Psychopathic Personality in Children: The Child Problematic Traits Inventory. J. Psychopathol. Behav. Assess..

[10] Hare R.D., Neumann C.S. (2008). Psychopathy as a Clinical and Empirical Construct. Annu. Rev. Clin. Psychol..

[11] Salekin R.T. (2017). Research Review: What do we know about psychopathic traits in children?. J. Child Psychol. Psychiatry.

[12] Frick P.J., Ray J.V., Thornton L.C., Kahn R.E. (2014). Can callous-unemotional traits enhance the understanding, diagnosis, and treatment of serious conduct problems in children and adolescents? A comprehensive review. Psychol. Bull..

[13] Lynam D.R., Caspi A., Moffitt T.E., Loeber R., Stouthamer-Loeber M. (2007). Longitudinal evidence that psychopathy scores in early adolescence predict adult psychopathy. J. Abnorm. Psychol..

[14] Colins O.F., Andershed H., Hellfeldt K., Fanti K. (2021). The incremental usefulness of teacher-rated psychopathic traits in 5- to 7-year olds in predicting teacher-, parent-, and child self-report antisocial behavior at six-year follow-up. J. Crim. Justice.

[15] Ezpeleta L., de la Osa N., Granero R., Penelo E., Domènech J.M. (2013). Inventory of callous-unemotional traits in a community sample of preschoolers. J. Clin. Child Adolesc. Psychol..

[16] López-Romero L., Colins O.F., Fanti K., Salekin R.T., Romero E., Andershed H. (2020). Testing the predictive and incremental validity of callous-unemotional versus the multidimensional psychopathy construct in preschool children. J. Crim. Justice.

[17] Haas S.M., Waschbusch D.A., Pelham W.E., King S., Andrade B.F., Carrey N.J. (2011). Treatment response in CP/ADHD children with callous/unemotional traits. J. Abnorm. Child Psychol..

[18] Hawes D.J., Dadds M.R. (2005). The treatment of conduct problems in children with callous-unemotional traits. J. Consult. Clin. Psychol..

[19] Waschbusch D.A., Walsh T.M., Andrade B.F., King S., Carrey N.J. (2007). Social problem solving, conduct problems, and callous-unemotional traits in children. Child Psychiatry Hum. Dev..

[20] Raine A. (2013). The Anatomy of Violence: The Biological Roots of Crime.

[21] Blair R., Salekin R.T., Lynam D.R. (2010). A cognitive neuroscience perspective on child and adolescent psychopathy. Handbook of Child and Adolescent Psychopathy.

[22] Frick P.J., Ray J.V., Thornton L.C., Kahn R.E. (2014). A developmental psychopathology approach to understanding callous-unemotional traits in children and adolescents with serious conduct problems. Child Psychol. Psychiatry.

[23] Kochanska G., Thompson R.A., Grusec J.E., Kuczynski L. (1997). The emergence and development of conscience in toddlerhood and early childhood. Parenting and Children’s Internalization of Values: A Handbook of Contemporary Theory.

[24] Aksan N., Kochanska G. (2005). Conscience in Childhood: Old Questions, New Answers. Dev. Psychol..

[25] Kochanska G., Aksan N., Ladd G.W. (2007). Conscience in childhood: Past, present, and future. Appraising the Human Developmental Sciences: Essays in Honor of Merrill-Palmer Quarterly.

[26] Thompson R.A., Newton E.K., Arsenio W.F., Lemerise E.A. (2010). Emotion in early conscience. Emotions, Aggression, and Morality in Children: Bridging Development and Psychopathology.

[27] Kochanska G. (1993). Toward a synthesis of parental socialization and child temperament in early development of conscience. Child Dev..

[28] Lykken D.T., Patrick C. (2006). Psychopathic personality: The scope of the problem. Handbook of Psychopathy.

[29] Calkins S.D., Blandon A.Y., Williford A.P., Keane S.P. (2007). Biological, behavioral, and relational levels of resilience in the context of risk for early childhood problems. Dev. Psychopathol..

[30] Kochanska G., Koenig J.L., Barry R.A., Kim S., Yoon J.E. (2010). Children’s conscience during toddler and preschool years, moral self, and a competent, adaptive developmental trajectory. Dev. Psychol..

[31] Dadds M.R., Allen J.L., Oliver B.R., Faulkner N., Legge K., Moul C., Woolgar M., Scott S. (2012). Love, eye contact, and the developmental origins of empathy v. psychopathy. Br. J. Psychiatry.

[32] Rutter M. (2012). Psychopathy in childhood: Is there a meaningful diagnosis?. Br. J. Psychiatry.

[33] Glenn A.L., Raine A., Venables P.H., Mednick S.A. (2007). Early temperamental and psychophysiological precursors of adult psychopathic personality. J. Abnorm. Psychol..

[34] Goffin K.C., Boldt L.J., Kim S., Kochanska G. (2018). A Unique Path to Callous-Unemotional Traits for Children who are Temperamentally Fearless and Unconcerned about Transgressions: A Longitudinal Study of Typically Developing Children from age 2 to 12. J. Abnorm. Child Psychol..

[35] Waller R., Wagner N.J., Barstead M.G., Subar A., Petersen J.L., Hyde J.S., Hyde L.W. (2020). A meta-analysis of the associations between callous-unemotional traits and empathy, prosociality, and guilt. Clin. Psychol. Rev..

[36] Blair R.J., Cipolotti L. (2000). Impaired social response reversal. A case of ‘acquired sociopathy’. Brain.

[37] Paciello M., Ballarotto G., Cerniglia L., Muratori P. (2020). Does the Interplay of Callous-Unemotional Traits and Moral Disengagement Underpin Disruptive Behavior? A Systematic Review. Adolesc. Health Med. Ther..

[38] Waller R., Gardner F., Hyde L.W. (2013). What are the associations between parenting, callous-unemotional traits and antisocial behavior in youth? A systematic review of evidence. Clin. Psychol. Rev..

[39] Backman H., Laajasalo T., Jokela M., Aronen E.T. (2021). Parental Warmth and Hostility and the Development of Psychopathic Behaviors: A Longitudinal Study of Young Offenders. J. Child Fam. Stud..

[40] Waller R., Gardner F., Viding E., Shaw D.S., Dishion T.J., Wilson M.N., Hyde L.W. (2014). Bidirectional associations between parental warmth, callous unemotional behavior, and behavior problems in high-risk preschoolers. J. Abnorm. Child Psychol..

[41] Mills-Koonce W.R., Willoughby M.T., Garrett-Peters P., Wagner N., Vernon-Feagans L. (2016). Family Life Project Key Investigators. The interplay among socioeconomic status, household chaos, and parenting in the prediction of child conduct problems and callous-unemotional behaviors. Dev. Psychopathol..

[42] Pasalich D., Dadds M., Hawes D., Brennan J. (2011). Callous-unemotional traits moderate the relative importance of parental coercion versus warmth in child conduct problems: An observational study. J. Child Psychol. Psychiatry.

[43] López-Romero L., Romero E., Gómez-Fraguela J.A. (2015). Delving into callous-unemotional traits: Concurrent correlates and early parenting precursors. J. Child Fam. Stud..

[44] Kimonis E.R., Bagner D.M., Linares D., Blake C., Rodriguez G. (2014). Parent training outcomes among young children with callous–unemotional conduct problems with or at risk for developmental delay. J. Child Fam. Stud..

[45] McDonald R., Dodson M.C., Rosenfield D., Jouriles E.N. (2011). Effects of a parenting intervention on features of psychopathy in children. J. Abnorm. Child Psychol..

[46] Kochanska G., Aksan N., Joy M.E. (2007). Children’s fearfulness as a moderator of parenting in early socialization: Two longitudinal studies. Dev. Psychol..

[47] Kochanska G. (1997). Multiple pathways to conscience for children with different temperaments: From toddlerhood to age 5. Dev. Psychol..

[48] Waller R., Trentacosta C.J., Shaw D.S., Neiderhiser J.M., Ganiban J.M., Reiss D., Leve L.D., Hyde L.W. (2016). Heritable temperament pathways to early callous-unemotional behaviour. Br. J. Psychiatry.

[49] Waller R., Shaw D.S., Hyde L.W. (2017). Observed fearlessness and positive parenting interact to predict childhood callous-unemotional behaviors among low-income boys. J. Child Psychol. Psychiatry.

[50] Waller R., Hyde L.W. (2018). Callous-unemotional behaviors in early childhood: The development of empathy and prosociality gone awry. Curr. Opin. Psychol..

[51] Colins O.F., Andershed H., Salekin R.T., Fanti K.A. (2018). Comparing different approaches for subtyping children with conduct problems: Callous-unemotional traits only versus the multidimensional psychopathy construct. Psychopathol. Behav. Assess..

[52] Frogner L., Gibson C.L., Andershed A.K., Andershed H. (2003). Childhood psychopathic personality and callous–unemotional traits in the prediction of conduct problems. J. Abnorm. Child Psychol..

[53] Lilienfeld S.O. (2018). The multidimensional nature of psychopathy: Five recommendations for research. Psychopathol. Behav. Assess..

[54] Salekin R.T., Andershed H., Batky B.D., Bontemps A.P. (2018). Are callous unemotional (CU) traits enough?. Psychopathol. Behav. Assess..

[55] Shaw D.S., Gilliom M., Ingoldsby E.M., Nagin S. (2003). Trajectories leading to school-age conduct problems. Dev. Psychol..

[56] Muratori P., Lochman J.E., Lai E., Milone A., Nocentini A., Pisano S., Righini E., Masi G. (2016). Which dimension of parenting predicts the change of callous unemotional traits in children with disruptive behavior disorder?. Compr. Psychiatry.

[57] Fleming G.E., Kimonis E.R., Niec L.N. (2018). PCIT for children with callous-unemotional traits. Handbook of Parent-Child Interaction Therapy.

[58] Young A.F., Powers J.R., Bell S.L. (2006). Attrition in longitudinal studies: Who do you lose?. Aust. N. Z. J. Public Health.

[59] Launes J., Hokkanen L., Laasonen M., Tuulio-Henriksson A., Virta M., Lipsanen J., Tienari P.J., Michelsson K. (2014). Attrition in a 30-year follow-up of a perinatal birth risk cohort: Factors change with age. PeerJ.

[60] World Health Organization (2016). Process of Translation and Adaptation of Instruments.

[61] Paterson G., Sanson A. (1999). The association of behavioural adjustment to temperament, parenting and family characteristics among 5-year-old children. Soc. Dev..

[62] Zubrick S.R., Lucas N., Westrupp E.M., Nicholson J.M. (2014). Parenting Measures in the Longitudinal Study of Australian Children: Construct Validity and Measurement Quality, Waves 1 to 4.

[63] López-Romero L., Domínguez-Álvarez B., Isdahl-Troye A., Romero E. (2021). Bidirectional Effects between Psychopathic Traits and Conduct Problems in Early Childhood: Examining Parenting as Potential Mediator. Rev. Psicol. Clínica Niños Adolesc..

[64] Rothbart M.K., Ahadi S.A., Hershey K.L. (1994). Temperament and social behavior in childhood. Merrill-Palmer Q..

[65] Rothbart M.K., Ahadi S.A., Hershey K.L., Fisher P. (2001). Investigations of temperament at 3–7 years: The Children’s Behavior Questionnaire. Child Dev..

[66] Dadds M.R., Hunter K., Hawes D.J., Frost A.D.J., Vassallo S., Bunn P., Merz S., El Masry Y. (2008). A measure of cognitive and affective empathy in children using parent ratings. Child Psychiatry Hum. Dev..

[67] López-Romero L., Maneiro L., Colins O.F., Andershed H., Romero E. (2019). Psychopathic traits in early childhood: Further multi-informant validation of the Child Problematic Traits Inventory (CPTI). J. Psychopathol. Behav. Assess..

[68] Muthen L., Muthen B. Mplus Version 7.4 Software. https://statmodel.com/.

[69] Marsh H.W., Wen Z., Hau K.T., Little T.D., Bovaird J.A., Widaman K.F. (2007). Unconstrained structural equation models of latent interactions: Contrasting residual-and mean-centered approaches. Struct. Equ. Modeling.

[70] Cham H., Reshetnyak E., Rosenfeld B., Breitbart W. (2017). Full information maximum likelihood estimation for latent variable interactions with incomplete indicators. Multivar. Behav. Res..

[71] Hu L.T., Bentler P.M. (1999). Cutoff criteria for fit indexes in covariance structure analysis: Conventional criteria versus new alternatives. Struct. Equ. Modeling.

[72] Salekin R.T., Lynam D.R. (2010). Handbook of Child and Adolescent Psychopathy.

[73] Colins O.F., Andershed H., DeLisi M. (2019). Childhood and adolescent psychopathy. Routledge International Handbook of Psychopathy and Crime.

[74] Ribeiro da Silva D., Rijo D., Salekin R.T. (2012). Child and adolescent psychopathy: A state-of-the-art reflection on the construct and etiological theories. J. Crim. Justice.

[75] Hyde L.W., Waller R., Trentacosta C.J., Shaw D.S., Neiderhiser J.M., Ganiban J.M., Reiss D., Leve L.D. (2016). Heritable and Nonheritable Pathways to Early Callous-Unemotional Behaviors. Am. J. Psychiatry.

[76] Viding E., Blair R.J., Moffitt T.E., Plomin R. (2005). Evidence for substantial genetic risk for psychopathy in 7-year-olds. J. Child Psychol. Psychiatry.

[77] Waller R., Wagner N. (2019). The Sensitivity to Threat and Affiliative Reward (STAR) model and the development of callous-unemotional traits. Neurosci. Biobehav. Rev..

[78] Domínguez-Álvarez B., Romero E., López-Romero L., Isdahl-Troye A., Wagner N.J., Waller R. (2021). A Cross-Sectional and Longitudinal Test of the Low Sensitivity to Threat and Affiliative Reward (STAR) Model of Callous-Unemotional Traits among Spanish Preschoolers. Res. Child Adolesc. Psychopathol..

[79] Dadds M.R., English T., Wimalaweera S., Schollar-Root O., Hawes D.J. (2019). Can reciprocated parent-child eye gaze and emotional engagement enhance treatment for children with conduct problems and callous-unemotional traits: A proof-of-concept trial. Child Psychol. Psychiatry.

